# COVID-rate: an automated framework for segmentation of COVID-19 lesions from chest CT images

**DOI:** 10.1038/s41598-022-06854-9

**Published:** 2022-02-25

**Authors:** Nastaran Enshaei, Anastasia Oikonomou, Moezedin Javad Rafiee, Parnian Afshar, Shahin Heidarian, Arash Mohammadi, Konstantinos N. Plataniotis, Farnoosh Naderkhani

**Affiliations:** 1 Concordia Institute for Information Systems Engineering, Concordia University, Montreal, QC Canada; 2 Department of Medical Imaging, Sunnybrook Health Sciences Centre, University of Toronto, Toronto, ON Canada; 3 Department of Medicine and Diagnostic Radiology, McGill University, Montreal, QC Canada; 4 Department of Electrical and Computer Engineering, Concordia University, Montreal, QC Canada; 5 Department of Electrical and Computer Engineering, University of Toronto, Toronto, ON Canada

**Keywords:** Medical imaging, Viral infection

## Abstract

Novel Coronavirus disease (COVID-19) is a highly contagious respiratory infection that has had devastating effects on the world. Recently, new COVID-19 variants are emerging making the situation more challenging and threatening. Evaluation and quantification of COVID-19 lung abnormalities based on chest Computed Tomography (CT) images can help determining the disease stage, efficiently allocating limited healthcare resources, and making informed treatment decisions. During pandemic era, however, visual assessment and quantification of COVID-19 lung lesions by expert radiologists become expensive and prone to error, which raises an urgent quest to develop practical autonomous solutions. In this context, first, the paper introduces an open-access COVID-19 CT segmentation dataset containing 433 CT images from 82 patients that have been annotated by an expert radiologist. Second, a Deep Neural Network (DNN)-based framework is proposed, referred to as the $\text {COVID-Rate}$, that autonomously segments lung abnormalities associated with COVID-19 from chest CT images. Performance of the proposed $\text {COVID-Rate}$ framework is evaluated through several experiments based on the introduced and external datasets. Third, an unsupervised enhancement approach is introduced that can reduce the gap between the training set and test set and improve the model generalization. The enhanced results show a dice score of 0.8069 and specificity and sensitivity of 0.9969 and 0.8354, respectively. Furthermore, the results indicate that the $\text {COVID-Rate}$ model can efficiently segment COVID-19 lesions in both 2D CT images and whole lung volumes. Results on the external dataset illustrate generalization capabilities of the $\text {COVID-Rate}$ model to CT images obtained from a different scanner.

## Introduction

Coronavirus disease 2019 (COVID-19) has been the world’s most threatening challenge of the twenty first century. According to the Coronavirus Resource Center of John Hopkins University (JHU)^1^, over 179 million confirmed cases of COVID-19, including over 3.8 million deaths, have been reported in 192 countries/regions by 24 June 2021. Numerous studies indicate that COVID-19 vaccination can effectively reduce disease transmission, hospital admissions, and deaths. However, due to new emerging COVID-19 variants^2–4^ the number of COVID-19 daily cases is still increasing in many areas. It is, therefore, crucial for healthcare professionals and authorities across the globe to find practical solutions to manage the COVID-19 pandemic and learn from this experience to be well prepared for potential future ones.

**Figure 1:**
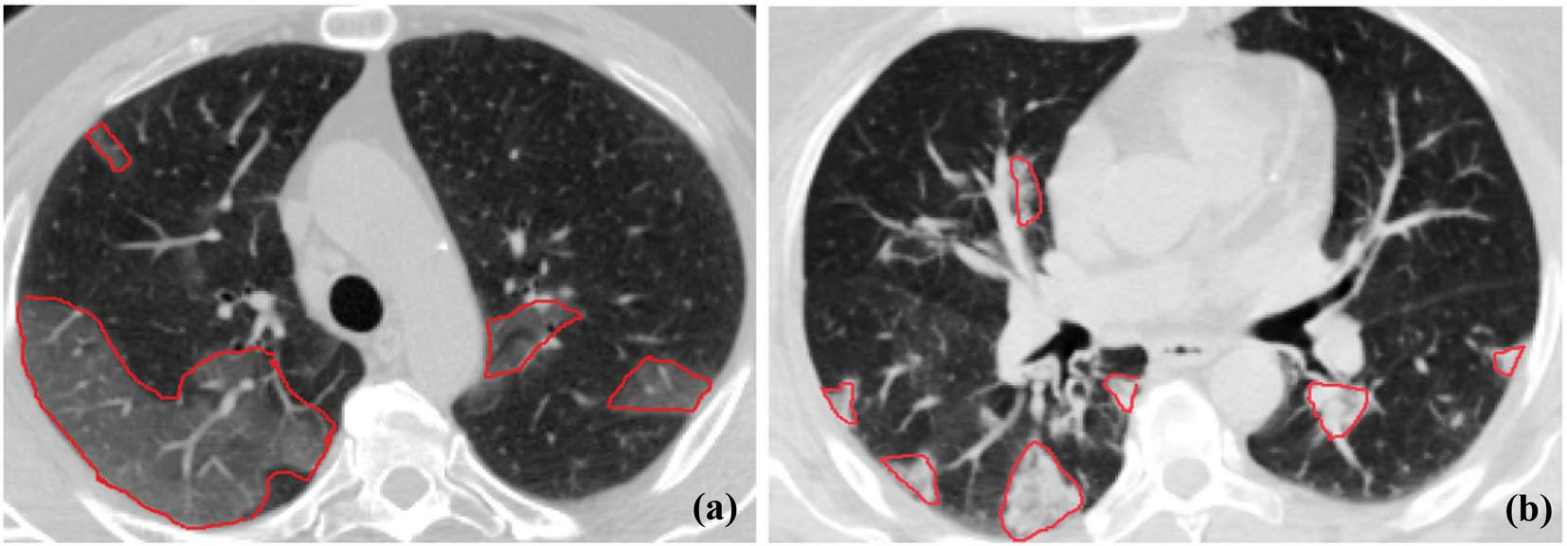
The most commonly observed CT patterns in COVID-19 pneumonia: (**a**) GGO pattern, and; (**b**) Consolidation pattern. Infection regions have been identified with red borders^13^.

Reverse Transcription-Polymerase Chain Reaction (RT-PCR) is the gold-standard test for the diagnosis of COVID-19. However, the RT-PCR test suffers from high false-negative rates and delayed results. Medical imaging, particularly Computed Tomography (CT) scans, has been recommended by the World Health Organization (WHO) as a complementary source of data for diagnosis and severity assessment of COVID-19. Countries with high rates of COVID-19 cases use chest CT images as the primary screening/monitoring technique. Several studies, therefore, have investigated COVID-19 manifestations on chest medical images. The most commonly observed chest imaging patterns in COVID-19 patients, as shown in Fig. 1, are Ground-Glass Opacity (GGO) and consolidation^5–7^. GGO refers to a slight increase in lung attenuation such that the underlying vessels are still observable^8^. The consolidation, on the other hand, is considered as a rise in lung intensity such that the underlying vessels are obscured^8^. The appearance of different types of imaging patterns and their location and distribution can be considered as specific signs of COVID-19 and provide helpful information for identifying the stage and severity of the disease^9–12^.

Evaluation and quantification of lung involvement in COVID-19 patients based on their chest images can help determine the disease stage, have an optimal allocation of the limited health resources, and make informed treatment decisions. Compared to other imaging modalities, CT imaging provides more accurate representations of COVID-19 lesions, making it the most informative imaging modality for the prognosis of COVID-19 pneumonia. Radiologists measure the COVID-19 lesions from the chest CT images and quantify the disease’s severity using different severity measures such as the Percentage of Opacity (PO) and CT severity score. The PO indicates the extent of involvement of the whole lung volume^14^, while the CT severity score is determined based on the spread of the COVID-19 lesions in each lobe^15^. During the pandemic era, when the number of patients is exponentially increasing, visual assessment and quantification of lung lesions by expert radiologists become expensive, laborious, and prone to error. Automatic segmentation of infectious regions can, therefore, help quantify the extent of lung involvement in patients confirmed with COVID-19, compute different severity scores, and speed up the treatment procedure.

**Contributions:** Motivated by the urgent quest to develop accurate and reliable automated models for prognostic assessment of COVID-19 pneumonia, we introduce an open-access COVID-19 segmentation dataset along with a Deep Learning (DL)-based framework for the segmentation of COVID-19 lung abnormalities from chest CT images. In summary, the main contributions of this study are as follows:

- We propose the $\text {COVID-Rate}$ framework, which is a DL-based model for segmenting COVID-19 lesions, including GGOs and consolidations. In the $\text {COVID-Rate}$ architecture, we incorporate multi-size kernels (ranging from 7 to 1) and dilated residual blocks in the encoding path to provide variable receptive fields for feature extraction. In addition, we implement a Squeeze-and-Excitation (SE) module to recalibrate channel-wise feature maps and improve model generalization. A convolution layer with a stride of two replaces the max-pooling layer to mitigate information loss during the down-sampling stage. Furthermore, we incorporate a context perception boosting module in the encoding path to learn multi-scale representations of COVID-19 manifestations using four parallel paths of dilated convolutions and a linear projection of the input feature maps. Implementing an un-symmetric network architecture leads to a greater emphasis on the mask reconstruction process. Adopting a hybrid loss function facilitates image-level and patch-level supervision during the training phase.
- We produced a high-quality COVID-19 segmentation dataset containing 433 annotated chest CT images from 82 COVID-19 patients, annotated by a thoracic radiologist with 20 years of experience.
- We introduce an unsupervised enhancement approach that can mitigate the gap between the training set and test set and improve model generalization on CT images obtained by a different scanner, addressing a critical challenge in applying AI in medical imaging.
- A novel synthetic data generation (augmentation) pipeline is proposed that generates synthetic pairs of CT images and infection masks by inserting the infectious regions from COVID-19 CT images into healthy CT images. The proposed method improves the model performance by introducing more variability to the training set.

Based on a set of comprehensive experiments, we have evaluated performance of the proposed $\text {COVID-Rate}$ model in segmenting COVID-19 lesions from 2D CT images and whole lung volumes. The above-mentioned contributions of the $\text {COVID-Rate}$ framework collectively have resulted in the state-of-the-art dice score of 0.8069 and specificity and sensitivity of 0.9969 and 0.8354, respectively. Additionally, experiments performed on the entire lung volumes indicate promising results, demonstrating that despite being trained only on infected CT images, the model can assist in patient-level lesion segmentation.

## Methods

**Figure 2:**
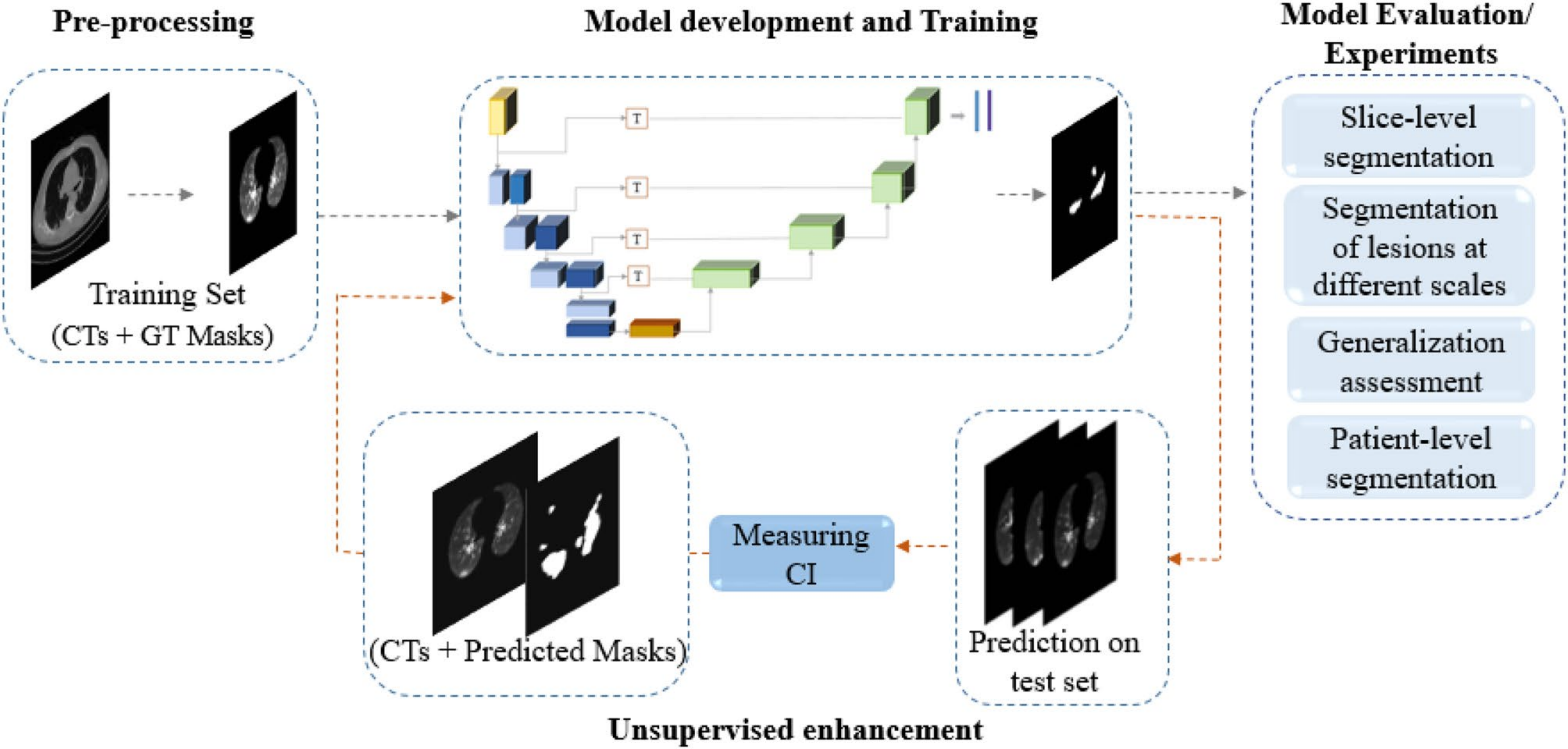
The overall pipeline of the proposed $\text {COVID-Rate}$ framework. GT and CI denote Ground Truth and Certainty Index, respectively.

In this section, we introduce the pixel-level labeled COVID-19 CT dataset along with the $\text {COVID-Rate}$ segmentation framework that takes thick-slice chest CT images of confirmed COVID-19 patients and automatically segments regions of COVID-19 infection. Figure 2 illustrates the overall pipeline of the $\text {COVID-Rate}$ framework, which consists of lung extraction and pre-processing stage, development and training stage, an unsupervised enhancement method, and a set of comprehensive experiments for model evaluation. Additionally, a novel synthetic data generation (augmentation) pipeline is introduced to tackle the issue of limited access to annotated training data.

### $\text {COVID-CT-Rate}$: pixel-level labeled COVID-19 CT dataset

As stated previously, DL-based segmentation networks need a large number of annotated CT images for efficient training. Most of the private annotated datasets, which are the basis of the existing COVID-19 lesion segmentation models, are not publicly available. Open-source pixel-level annotated datasets can promote developing DL networks for COVID-19 lesions segmentation. Furthermore, evaluation of segmentation models on public datasets makes it feasible to fairly compare different segmentation models. To address the aforementioned issues, via this manuscript, we introduce a pixel-level annotated CT dataset, referred to as the $\text {COVID-CT-Rate}$. The CT images used to generate the COVID-19 segmentation dataset are a subset of our previous COVID-19 diagnosis CT dataset^16^, which is accessible through Figshare^16^. A SIEMENS, SOMATOM Scope scanner has been used to obtain all axial CT images in $\text {COVID-CT-Rate}$. All CT images are reconstructed by the Filtered Back Projection method using the reconstruction matrix size of $512 \times 512$ and D40s kernel, which modifies the data frequency contents and mitigates the noise. All CT images are obtained without contrast enhancement and saved in the Digital Imaging and Communications in Medicine (DICOM) format and the Hounsfield Unit^16^.

**Figure 3:**
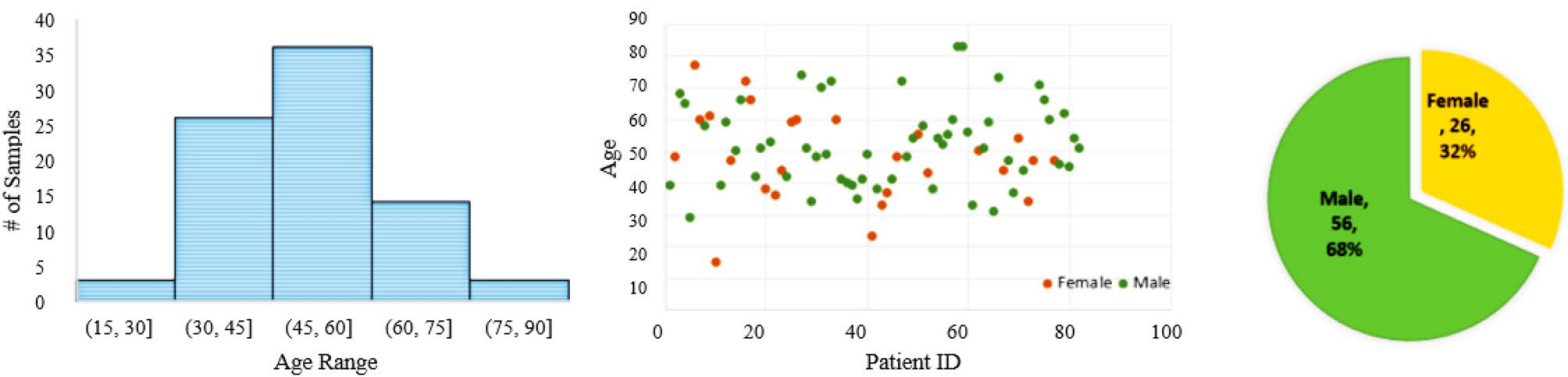
Age and gender distribution of the $\text {COVID-CT-Rate}$ dataset.

In the annotation process, a standard U-Net model is firstly trained on an open-access COVID-19 segmentation dataset^17^. The trained model then takes the CT images as unseen test sets and predicts the infection masks. Next, a thoracic radiologist with 20 years of experience in lung imaging, carefully modified and verified the generated infection masks. Overall, we annotated 433 CT images from 82 COVID-19 patients. The patients’ average age is $51 \pm 3.59$ years (mean ± std). Figure 3 represents the age and gender distribution of the COVID-19 patients in the $\text {COVID-CT-Rate}$. As illustrated in Fig. 3, the COVID-CT-Rate is gender-imbalanced, i.e., the proportion of males in this dataset is more significant than females. However, it is worth mentioning that the same pattern applies to several datasets pertaining to COVID-19^18^, probably because men are more vulnerable to the disease than women^19^. Besides, clinical findings indicate no association between CT scores and patients’ gender in COVID-19 pneumonia^15^.

**Figure 4:**
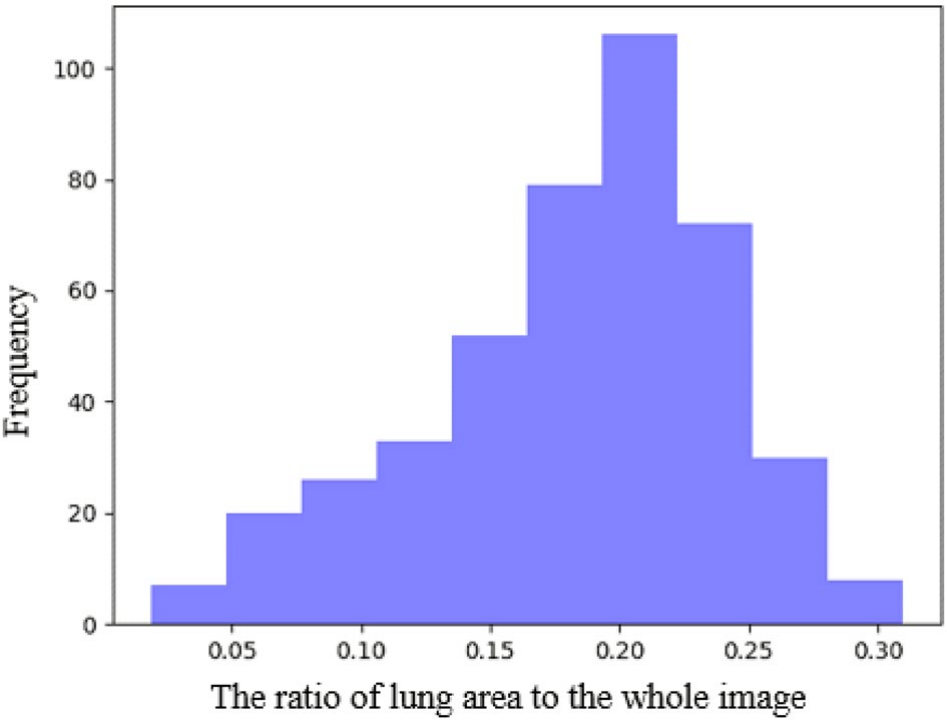
The distribution of the lung area to the image area in COVID-CT-Rate dataset.

**Figure 5:**
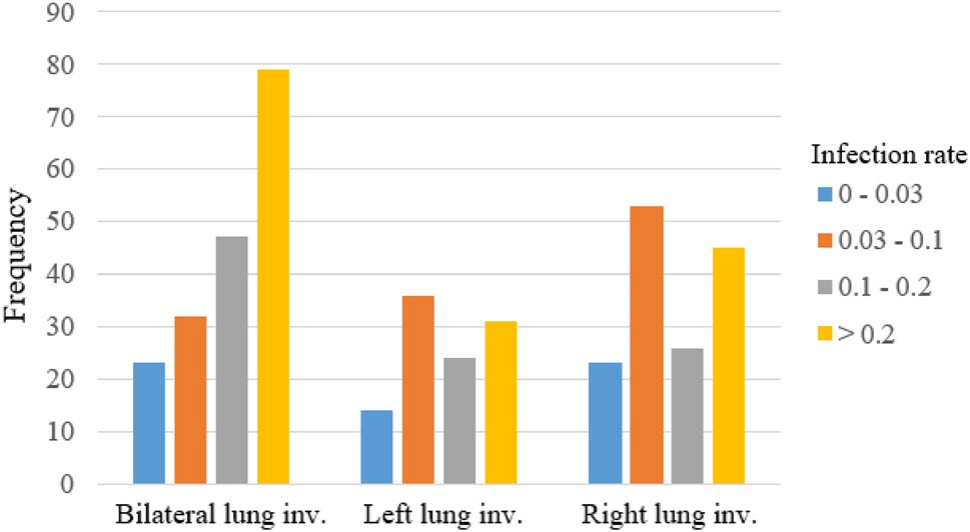
The distribution of slice-level infection rate in COVID-CT-Rate images with bilateral, left, and right lung involvement.

**Figure 6:**
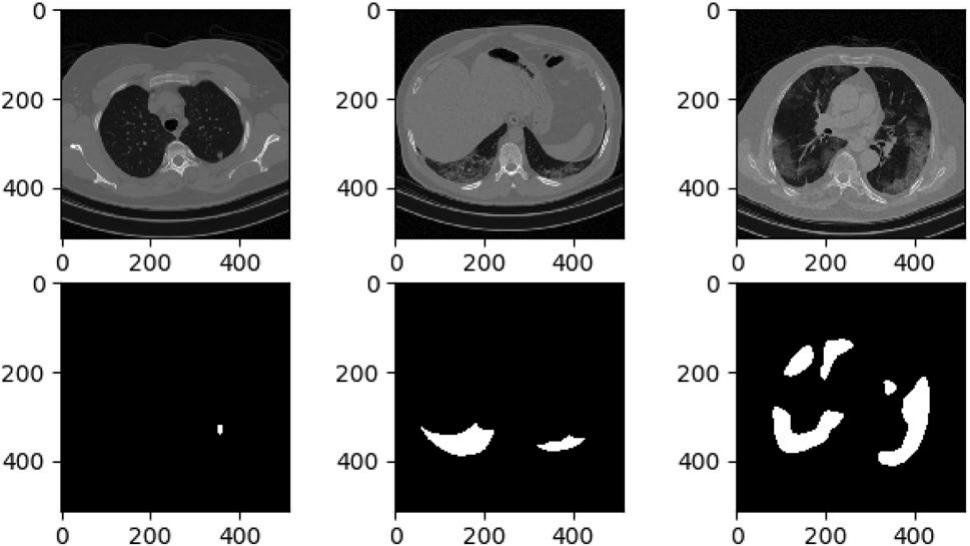
Examples of CT images with various infection rates and different positions in lung volume. First row: CT images. Second row: Ground truth masks.

COVID-19 CT findings demonstrate that most COVID-19 patients have “bilateral” lung involvement, meaning that the disease affects both the right and left lungs. The COVID-CT-Rate dataset illustrates a similar behavior, i.e., 42% of CT images display bilateral lung involvement, 34% include lesions in the right lung, and the remaining 24% involve the left lung. The CT images are from diffident parts of the lung (top, middle, and bottom) with different infection rates. The distribution of lung area to the whole image area in COVID-CT-Rate is shown in Fig. 4. More specifically, we selected 73% of the CT images from the middle part of the lung and the remaining 27% from the top and bottom sections to cover different appearances of the lung region in a CT image. Figure 5 illustrates the distribution of infection rate in CT images with bilateral, left, and right lung involvements. The infection rate in CT images with left/right lung involvement is obtained by dividing the infection region area by the left/right lung region. In CT images with bilateral lung involvement, the infection rate is calculated by dividing the area of total infection region by the whole lung area in that CT image. These considerations support the AI model to achieve better performance when predicting the infection regions on a whole lung volume. GGOs and consolidations, as the most prevalent manifestations of COVID-19, have been annotated in our dataset. Examples of CT images with their ground truth masks are shown in Fig. 6.

### COVID-19 lesion segmentation network

**Figure 7:**
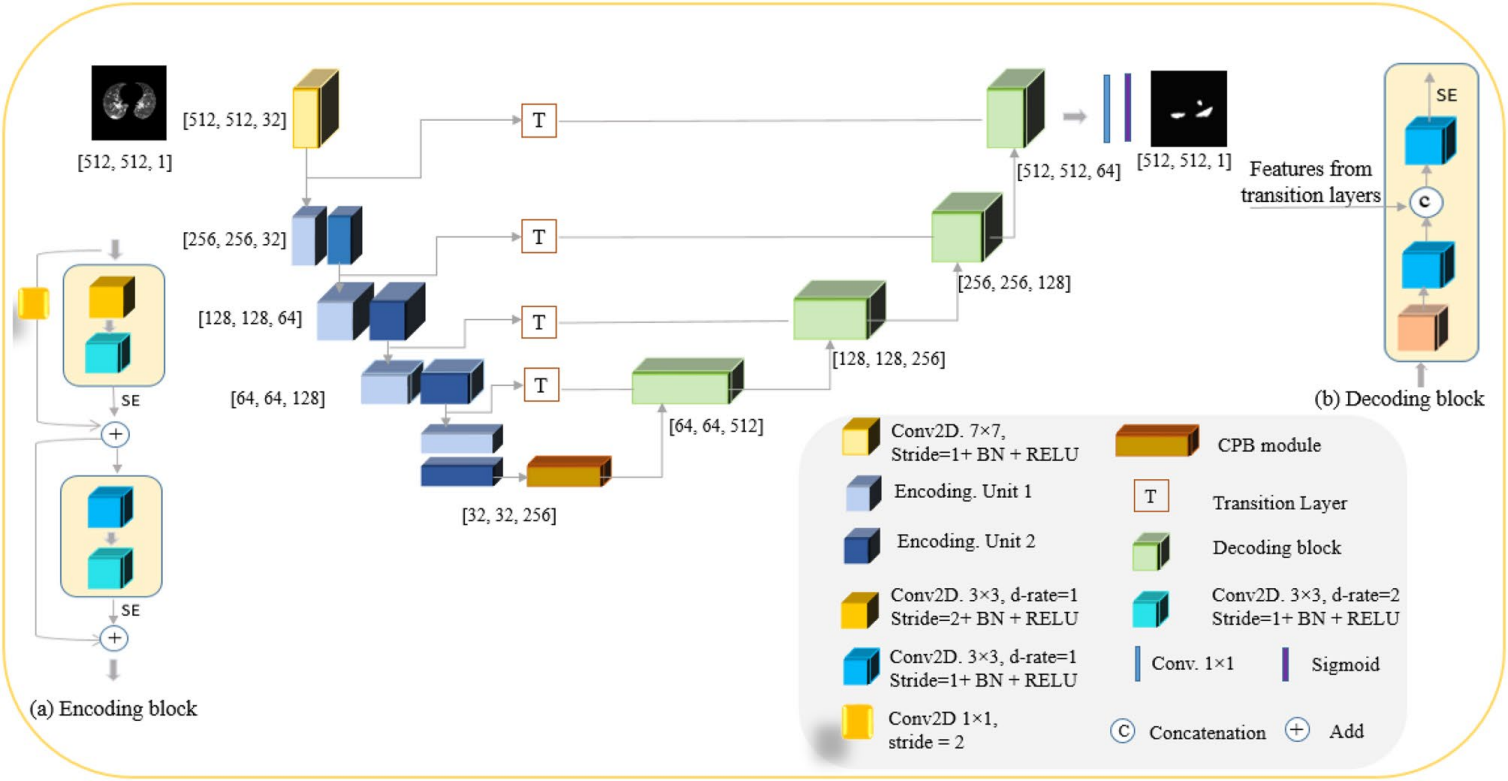
Architecture of the proposed $\text {COVID-Rate}$ segmentation framework.

The architecture of the proposed $\text {COVID-Rate}$ framework (as shown in Fig. 7) is an encoder-decoder-based network containing an encoding path, transition layers, a context perception boosting module, and a decoding path. These four underlying components are described below:

*Encoding Path* extracts informative features from chest images. The Encoding Path is initiated with a $7 \times 7$ convolution layer with 32 filters, followed by a Batch Normalization (BN) layer and ReLU activation function. Four encoding blocks with 32, 64, 128 and 256 filters, with the architecture represented in Fig. 7(a), are then applied sequentially. The encoding block has two units, each consisting of two successive $3 \times 3$ convolution layers with dilation rates equal to 1 and 2, followed by the BN layer and ReLU function. The first convolution layer of the first unit in the encoding block uses a stride of 2 for down-sampling. The max-pooling layer is replaced with a convolution layer with a stride of two to mitigate information loss during down-sampling. The output of each unit is fed into a Squeeze-and-Excitation (SE) module^22^ to recalibrate channel-wise feature maps and improve model generalization. The input feature maps of each unit are then added to the output feature maps of SE module using element-wise addition operation. Transmitting the feature maps to the deeper levels (residual linking) facilitates the convergence of the network and alleviates the gradient vanishing issue^20^.

**Figure 8:**
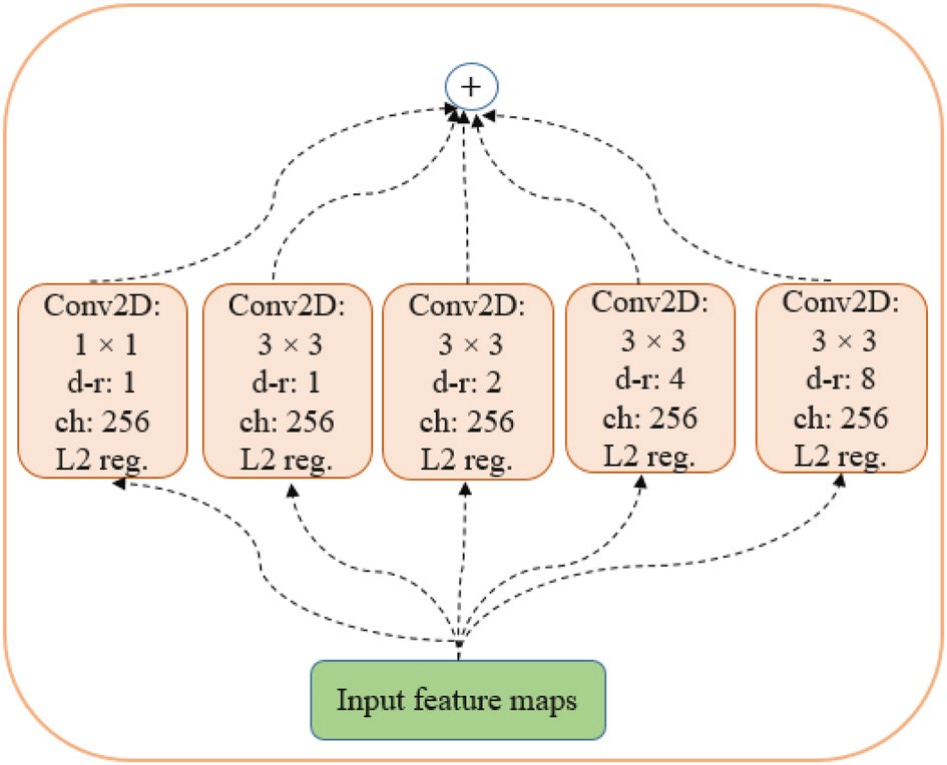
Architecture of the proposed context perception boosting module.

*Context Perception Boosting (CPB) Module*: As mentioned previously, a critical challenge in segmenting COVID-19 lung abnormalities is various sizes and scattered distribution of the regions of infection. Since a Convolutional Neural Networks (CNN)-based encoder extracts the information from a local area of the global image by applying the convolution kernel, it may fail to learn the long-range dependencies of the global image. To address this problem and inspired by^21^, we adopt a context perception boosting module in the last encoding block of the $\text {COVID-Rate}$ network. This module, as demonstrated in Fig. 8, contains a $1 \times 1$ convolution layer for linear projection of the input feature maps and four parallel $3 \times 3$ convolution layers with different dilation rates. *L*2 regularization is applied after each convolution operation to penalize weight matrices and mitigate over-fitting. The outputs of different convolution layers are then integrated using element-wise addition. The dilation rates for parallel convolutions are set to 1, 2, 4 and 8. The context perception boosting module increases the receptive field of the extracted feature maps by incorporating multi-scale kernels, helping the network detect COVID-19 abnormalities of various sizes.

*Transition layer*: A critical feature of the U-Net, that has also been leveraged in many other segmentation networks, is using skip connections to transfer encoding feature maps with higher spatial information to the decoding blocks. Here, we apply a transition layer, a $1 \times 1$ convolution layer followed by ReLU activation, on extracted features from the encoding path before concatenating them with the decoding feature maps. This transition layer enriches the spatial context information of encoding features, particularly those from primary encoding blocks, elevating their contribution to the mask reconstruction.

*Decoding Path* takes the extracted features of the context perception boosting module as input, aiming to restore the spatial representation and generate masks indicating the regions of infection. For reconstructing precise infection masks, four decoding blocks are adopted within the decoder path of the $\text {COVID-Rate}$ segmentation network. Each decoding block, presented in Fig. 7(b), starts with an up-sampling layer to expand the feature maps’ size to the size of encoding feature maps. The up-sampling layer’s outputs are fed to a $3 \times 3$ convolution layer followed by a BN layer, and a ReLU activation function and then concatenated to the extracted features from the transition layer. A $3 \times 3$ convolution layer followed by a BN layer, and a ReLU layer is then applied to the skip connection’s output to learn the spatial representation of the contextual information. The numbers of filters for decoding blocks are 512, 256, 128 and 64. The first decoding block of the $\text {COVID-Rate}$ network has the maximum number of filters. Indeed, the number of feature channels in each decoding block is higher than its corresponding encoding block, leading to a greater emphasis on the mask reconstruction process. Each encoding block’s output is fed into a SE module^22^ to calibrate channel-wise features. The output of the last decoding block has the same spatial dimension as the original CT images. A $1 \times 1$ convolution layer with one feature channel is applied on the last decoding block’s output. The sigmoid activation function predicts the probability of each pixel belonging to the infection class.

### Unsupervised enhancement

Different types of scanners and image acquisition settings produce CT images with heterogeneous characteristics, resulting in poor performance of a trained deep learning-based model on unseen CT images. To address this critical challenge, we propose an unsupervised enhancement method for segmentation networks, illustrated in Fig. 2, to reduce the shift between the training set and different test sets and elevate the model’s generalization on various test sets. In other words, our method automatically extracts and annotates a portion of a test set using a probabilistic selection metric. The selected data points and their associated predicted masks are then leveraged to re-train and enhance the initially trained model. For this purpose, we extract a subset of the test set for which the model predicts the most confident masks. Assuming that we are blind to the lesion masks of the test set, we introduce a Certainty Index (CI) to measure the model’s confidence on test images’ predicted masks, which is defined as follows

1 $$\begin{aligned} CI ({\hat{Y}}) = \frac{\sum _{i=1}^{N} {P_i}}{N}\quad for\quad P_i > Cut-off\,threshold, \end{aligned}$$

where ${\hat{Y}}$ represents the predicted masks on a test CT image, $P_i$ is the predicted probability of pixel *i*, and *N* denotes the number of pixels for which $P_i$ is more significant than a cut-off threshold. In a binary predicted mask, the pixels with $P_i$ less than the cut-off threshold are considered as the background class, and the rest are assigned to the foreground class. Indeed, the CI determines the average of $P_i$ over pixels belonging to the lesion regions, measuring the model confidence in segmenting lesion regions in the minority class. Next, we sort the predicted masks by the value of the CI and select the top 25%, if their measure CI is not less than 90%, as the most certain predictions. It should be noted that the values of 25% and 90% have been determined based on the test sets’ sizes and measured CIs in our experiments. Next, we utilize the subset of the most-confident CT images with their predicted lesion masks to retrain our initial trained model. In the first experiment, we examined the proposed method upon a set of CT images obtained from the same scanner as the COVID-CT-Rate dataset for which we did not have the ground truth masks and re-evaluated the performance of the retrained model on our test set. In another experiment, we first evaluated our trained model on a public dataset independent of our training set. Then, we applied the proposed unsupervised enhancement method to select the most certain predictions and retrain the model. The performance of the retrained model was evaluated on the same public dataset containing both infected and non-infected CT images. In both cases, the model yielded improved results. However, the results on the public dataset showed more improvement. The reason is that, on the first test, the model had previously seen the CT images from that scanner, and the retraining process on the most-certain predictions with the predicted infection masks could not give much information to the model. In contrast, the model in the second experiment didn’t have any information about the new data acquired by a different scanner. Consequently, through the retraining process, the model was able to learn helpful information about the specifications of the unseen images, which led to further improvements. In other words, the proposed unsupervised method could mitigate the gap between the training set and the independent set, resulting in an enhanced generalization.

### Synthetic data generation specific to COVID-19 lesion segmentation

Using data augmentation techniques^23,24^ has become a standard solution in different computer vision tasks to tackle the lack of sufficient training data and prevent over-fitting. These techniques mainly include applying simple transformations such as rotating, horizontal/vertical flip, zooming, and/or translation on original images. However, these transformations make minor changes to the existing images, resulting in limited diversity on newly generated images. Development of new data augmentation techniques is therefore crucial to introduce more variability to the synthetic samples for better data reinforcement. For example, recently, Ref.^25^ implemented a specific data augmentation approach into a generative adversarial segmentation network and achieved improved results. Capitalizing on this vision, we propose a specific approach for generating synthetic pairs of CT images and their corresponding infection masks by extracting the COVID-19 regions of infection from infected chest CT images and inserting them into healthy chest CT images. We use a chest CT image containing COVID-19 regions of infection alongside its infection mask and a chest CT image from a healthy person acquired by the same scanner alongside its lung mask as the input. First, CT images are normalized using the mean and standard deviation of their pixel intensities. Next, regions of infection are extracted from the COVID-19-infected CT image using the infection mask. Lung regions from the normalized healthy CT image are extracted using its corresponding lung mask. Then, the location of the region of infection is evacuated by element-wise multiplication of normalized healthy lung regions and the inverted infection mask. In the next step, the regions of infection are added to the evacuated healthy CT image. Finally, the regions of infection outside the healthy lung regions are trimmed off using the healthy lung mask, and the new infected CT image is generated. The synthetic infection mask is adjusted for the synthetic infected CT image by multiplying the healthy lung mask by the infection mask.

### Hybrid loss function

For a segmentation task, when dealing with highly imbalanced datasets, which is common when dealing with COVID-19 chest CT images, equally penalizing False Negatives (FNs) and False Positives (FPs) during the training process will result in a low sensitivity in predicted masks. To tackle this problem, we trained the $\text {COVID-Rate}$ model by minimizing a hybrid loss function over the training epochs to supervise the model in both image-level and local areas. The hybrid loss is a combination of weighted Binary Cross-Entropy (w-BCE)^26^ and Focal Tversky Loss (FTL)^27^ and is given by

2 $$\begin{aligned} L_{Total}=L_{w-BCE} +\kappa \,L_{TFL}, \end{aligned}$$

where $\kappa$ determines the FTL’s contribution to the total loss function and is set to 1 in our experiments. The FTL, which is a generalization of the Tversky Index (TI)^28^, is a loss function that improves the model performance by assigning higher weights to hard pixels and has been introduced explicitly for the segmentation of class-imbalanced datasets. The TI and FTL are defined as follows

3 $$\begin{aligned} TI= \frac{{\sum _{i=1}^{N}}{P_{li}\,GT_{li}}}{{\sum _{i=1}^{N}}{P_{li}\,GT_{li}}+\alpha {\sum _{i=1}^{N}}{P_{li}\,GT_{bi}}+\beta \,{\sum _{i=1}^{N}}{P_{bi}\,GT_{li}}} \end{aligned}$$

4 $$\begin{aligned} FTL= & {} \sum _c{(1 - TI)}^\frac{1}{\gamma } \end{aligned}$$

where $P_{li}$ is the probability that pixel *i* belongs to the lesion class and $P_{bi}$ is the probability that pixel *i* is of the background class. The $GT_li$ is one for a pixel labeled as lesion and zero for a pixel labeled as background, and vice versa for the $GT_bi$. The trade-off between FPs and FNs can be adjusted through the hyper-parameters $\alpha$ and $\beta$. Term $\gamma$ is defined within the range of [1, 3] and forces the model to detect small Regions of Interest (RoI). We set $\alpha = 0.7$, $\beta = 0.3$, and $\gamma = \frac{4}{3}$^27^. Compared to the standard $L_{BCE}$, the $L_{w-BCE}$ can improve the model’s performance by assigning higher weights for the target pixels (i.e., COVID-19 lesions) instead of weighting all pixels equally.

### Experiments

This research work is performed based on the policy certification number 30013394 of Ethical acceptability for secondary use of medical data approved by Concordia University, Montreal, Canada. All experiments were conducted in accordance with the Tri-Councel Policy Statement of Ethical Conduct for Research Involving Humans in Canada. Furthermore, informed consent is obtained from all the patients. To evaluate the efficacy and potential limitations of the proposed $\text {COVID-Rate}$ framework, we performed an extensive set of experiments. In particular, we have investigated the model’s capability in detecting regions of infection of different sizes on the CT images. The conducted experiments analyze the model’s performance in segmenting COVID-19 lesions in: (i) Slice-level segmentation, where only CT images containing COVID-19 abnormalities are fed to the network, and; (ii) Patient-level experiments when the model is tested on the whole CT volume containing CT images with and without COVID-19 lesions. To explore $\text {COVID-Rate}$’s generalization, we experimented on an independent dataset and evaluated the model’s performance on CT images acquired by a different scanner in a different imaging center. We also tested the $\text {COVID-Rate}$ on CT volumes of 50 COVID-19 patients to assess performance of the model in discriminating infected CT images from non-infected ones.

#### Description of datasets

Four datasets are used in different steps of the experiments as outlined below:

- ***Dataset A***^17^, which is a public dataset containing chest CT volumes of 10 COVID-19 patients, a total of 2, 581 CT images. The matrix size of the images is $512\times 512$ pixels. In total, 1351 CT images (out of 2581) have shown COVID-19 manifestations and have been annotated by three expert radiologists. The segmentation of lung regions exists for all CT slices.
- ***Dataset B (the ***$\text {COVID-CT-Rate}$ ***dataset)***: The in-house dataset, which contains 433 annotated CT images from 82 COVID-19 patients.
- ***Dataset C***^29^, which contains nine COVID-19 chest CT volumes, a total of 829 CT slices. In total, 373 out of 829 CT images indicated COVID-19 lesions and have been segmented by a radiologist. The segmentation of lung regions has been performed for the whole CT volumes. The CT slices have a dimension of $630\times 630$ pixels and are resized to $512\times 512$ pixels for our experiments.
- ***Dataset D***, introduced in our previous work^16^, containing chest CT volumes from 76 healthy cases, 171 COVID-19 patients, and 60 patients with other types of Community Acquire Pneumonia (CAP). In this dataset, 55 out of 171 COVID-19 CT volumes have slice-level labels, indicating which CT slices demonstrate COVID-19 lesions. The CT images used for the $\text {COVID-CT-Rate}$ dataset are a subset of COVID-19 cases in Dataset D.

The first two datasets (Dataset A and B) are used for training/testing purposes, while the third and fourth datasets (Dataset C and D) are used to evaluate the model’s performance from various aspects.

#### Evaluation metrics

We evaluate the $\text {COVID-Rate}$ model’s performance in segmenting COVID-19 lesions by comparing the predicted segmentation masks with the ground truth labels. The following metrics are used for evaluation purposes:

***Dice Similarity Coefficient (DSC)***, which is the most commonly used metric for segmentation. The DCS measures the relative overlap of the predicted regions of infection and their ground truth labels. It takes the value one as its maximum when the two regions have a complete agreement and a minimum value of zero when there is no overlap between the two underlying areas. The DSC is defined as follows

5 $$\begin{aligned} \text {DSC} = \frac{2 (|Pr| \cap |GT|)}{|Pr| + |GT|}, \end{aligned}$$

where terms *Pr* and *GT* correspond to the set of pixels belonging to the predicted and the ground truth regions, respectively. The symbol $\cap$ represents the intersection operation, and $|\cdot |$ is the cardinality operator.

***Sensitivity (SEN) and Specificity (SPC)***: The SEN metric calculates the number of pixels correctly labeled as COVID-19 lesions in the predicted masks relative to the total number of pixels identified as COVID-19 abnormalities in the ground truth. The SPC metric measures the number of pixels correctly labeled as the background class relative to the total number of background pixels in the ground truth. SEN and SPC metrics are defined as follows

6 $$\begin{aligned} SEN= & {} \frac{TP}{TP + FN},\end{aligned}$$

7 $$\begin{aligned} \text {and} \quad SPC= & {} \frac{TN}{TN + FP}, \end{aligned}$$

where *TP*, *FN*, *TN*, and *FP* are the number of pixels in the true positive, false negative, true negative, and false-positive regions, respectively.

***Mean Absolute Error (MAE)***, which calculates the absolute error between each pixel’s predicted and ground-truth label and takes the average over the whole pixels. The MAE is given by

8 $$\begin{aligned} MAE = \frac{1}{w \times h}{\sum _{j=1}^{w}} {\sum _{i=1}^{h}} { \big |Pr_{ij} - GT_{ij}\big |}. \end{aligned}$$

The MAE metric has a minimum of zero if all the pixels are correctly labeled and a maximum of one when all the pixels are predicted with the wrong label.

#### Pre-processing step

In the pre-processing step, we extract lung regions from all CT images using a lung segmentation model, referred to as the “U-net (R231CovidWeb)”^30^. This model has been trained and evaluated on three public datasets covering six different types of lung diseases and then fine-tuned on a small COVID-19 dataset. The U-net (R231CovidWeb) can accurately extract lung regions from COVID-19 CT images and has already been used for calculating lung area in COVID-19 outcome prediction^31,32^, as a pre-processing step in COVID-19 diagnosis^33,34^, and in lesion segmentation studies^35,36^. It is worth mentioning that segmenting lung abnormalities can also be performed in an end-to-end fashion without extracting lung regions via a pre-processing step. Removing non-lung regions via a pre-processing step, however, enables the learning model to be restricted to the region of interest, reducing false-positive detection outside the lung area. Furthermore, rendering all non-lung pixels to zero decreases the computational costs and helps the model converge faster. For these reasons, several research studies with the objective of COVID-19 diagnosis or lesion segmentation from CT images have extracted lung regions in their pre-processing phase. After segmenting the lung regions, only CT images containing the lung tissues are passed to the next stage, and the rest are eliminated. Each CT image is normalized based on its mean and standard deviation (std). The datasets are divided into three independent groups for training (60%), validation (10%), and testing (30%). To avoid information leakage, we kept the underlying datasets patient-independent, meaning that patients’ CT images are not shared between training, validation, and test sets. Furthermore, to improve the model performance on unseen data and mitigate over-fitting issues, we use real-time data augmentation strategies, including zooming, shifting, and shearing, where artificial images are synthesized from each mini-batch of original CTs during the training process. The model observes each synthetic image only once, resulting in an enhancement in the model’s overall generalization ability. The network uses the Adam optimizer with an initial learning rate of 0.001 over 100 epochs and will stop if the loss function on the validation set does not decrease over ten epochs.

#### Quantitative and qualitative analysis

We use a combination of Datasets A and B, i.e., a total of 1784 CT images with COVID-19 manifestations from 92 patients to train/test the proposed $\text {COVID-Rate}$ model. The average of the evaluation metrics calculated on the whole test set over 10-fold cross-validation is represented in Table 1. The average DSC over the entire test set over 10-fold cross-validation is equal to 0.8036. The average MAE is 0.0053, which means that 99.47% of pixels on the test set have been labeled correctly. The average of SPC and SEN metrics are 0.9968 and 0.834, respectively. The calculated standard deviations for the evaluation metrics demonstrate that the different folds’ results do not vary significantly, indicating that the model has a reliable performance on different test sets of the 10-fold cross-validation.

**Table 1:** Performance of the proposed segmentation network on the test set through 10-fold cross-validation approach.

	DSC	SPC	SEN	MAE
Ave ± std	$0.8036 \pm 0.033$	$0.9968 \pm 0.0007$	$0.834 \pm 0.044$	$0.0053 \pm 0.001$

We assess effects of different loss functions, including weighted binary cross-entropy (w-BCE), FTL, and the hybrid loss function on the model performance; results are shown in Table 2. The FTL loss function improves the model performance (DCS, SPC, and MAE metrics) compared to the w-BCE loss function. However, it decreases the model sensitivity from 83.94 to 82.58%. The model trained on the hybrid loss function slightly reduces the SEN compared to the w-BCE loss but improves DSC, SPC, and MAE evaluation metrics.

**Table 2:** Quantitative evaluation of our proposed model trained with different loss functions.

Loss function	DSC	SPC	SEN	MAE
w-BCE	0.7810	0.9960	**0**.**8394**	0.0056
FTL	0.7935	0.9967	0.8258	0.0054
Hybrid loss function	**0**.**8036**	**0**.**9968**	0.8340	**0**.**0053**

The presented results are the average of the obtained results through a 10-fold cross-validation process. Significant values are in bold.

**Figure 9:**
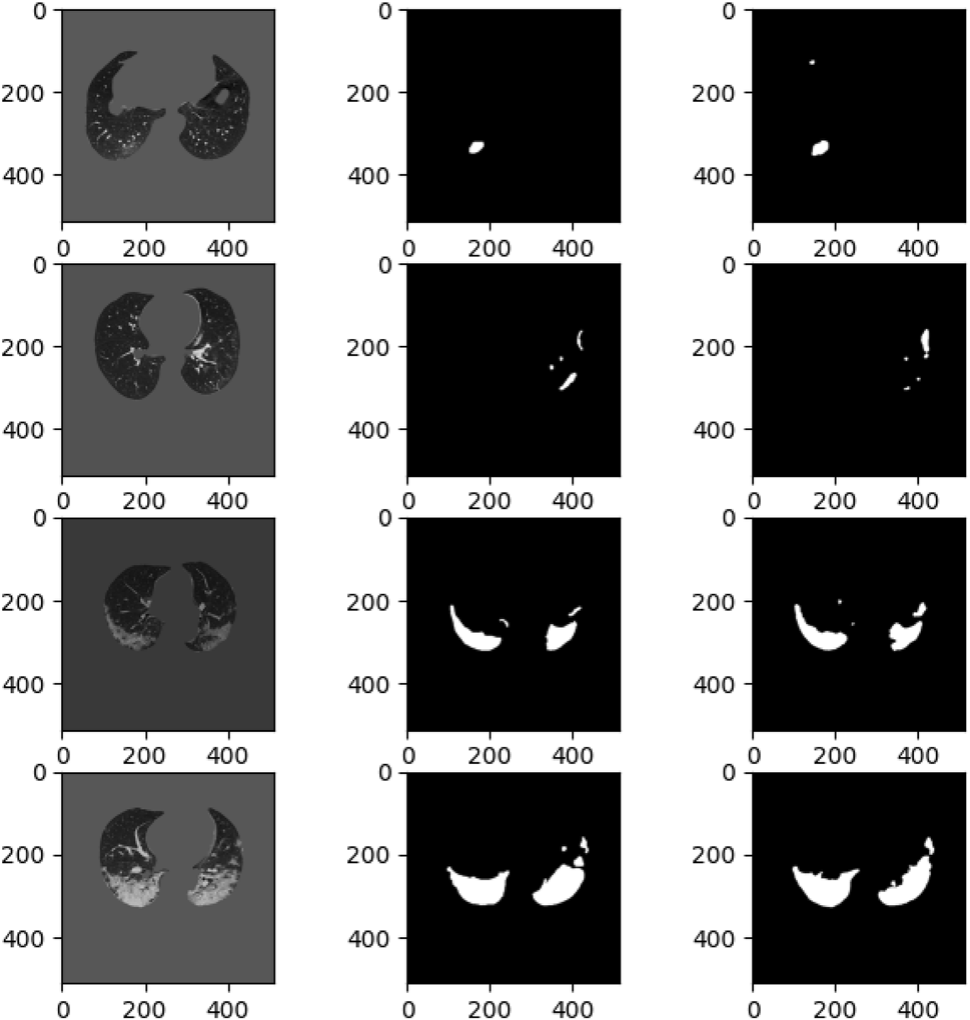
Qualitative evaluation of model performance on test set. From left: Original image, Ground truth mask, Predicted mask. Row 1 and 2: Test images from group A with infection rate less than 0.015, Row 3 and 4; Test images from group B with infection rate more significant that 0.015.

To better investigate our model’s performance in segmenting COVID-19 lesions at different scales, we categorize the test images into two groups: (i) *Group-A*, CT images with infection rate smaller than 0.015, and; (ii) *Group-B*, CT images with the infection rate larger than 0.015. The infection rate is calculated by dividing COVID-19 lesions’ area by the lungs’ area in a CT slice. Table 3 represents performance of the $\text {COVID-Rate}$ model across these two groups, indicating the median of evaluation metrics and their Inter-quartile Ranges (IQR) for 25% and 75% percentiles. As can be seen, although the model yields better results on CT images in Group-B, its performance in dealing with small regions of infection is also acceptable. The average DCS for Group-A (median: 0.6671, IQR: 0.403–0.7877) and Group-B (median: 0.8264, IQR: 0.7421–0.8754) are calculated. The quantitative analysis over Group-A and Group-B indicates that the proposed model is reliable in segmenting COVID-19 lesions at different scales. To better understand the model’s capabilities, we visualized some examples of Group-A and Group-B along with their predicted masks in Fig. 9.

**Table 3:** Performance of the proposed $\text {COVID-Rate}$ network on CT images of groups A and B. The presented results are the average of the 10-fold cross-validation.

	Group A	Group B
	Median (IQR 25%, IQR 75%)	Median (IQR 25%, IQR 75%)
DSC	0.6671 (0.403, 0.7877)	0.8264 (0.7421, 0.8754)
SPC	0.9997 (0.9994, 0.9998)	0.9979 (0.9965, 0.9990)
SEN	0.7689 (0.4182, 0.9434)	0.8969 (0.7637, 0.9578)

**Figure 10:**
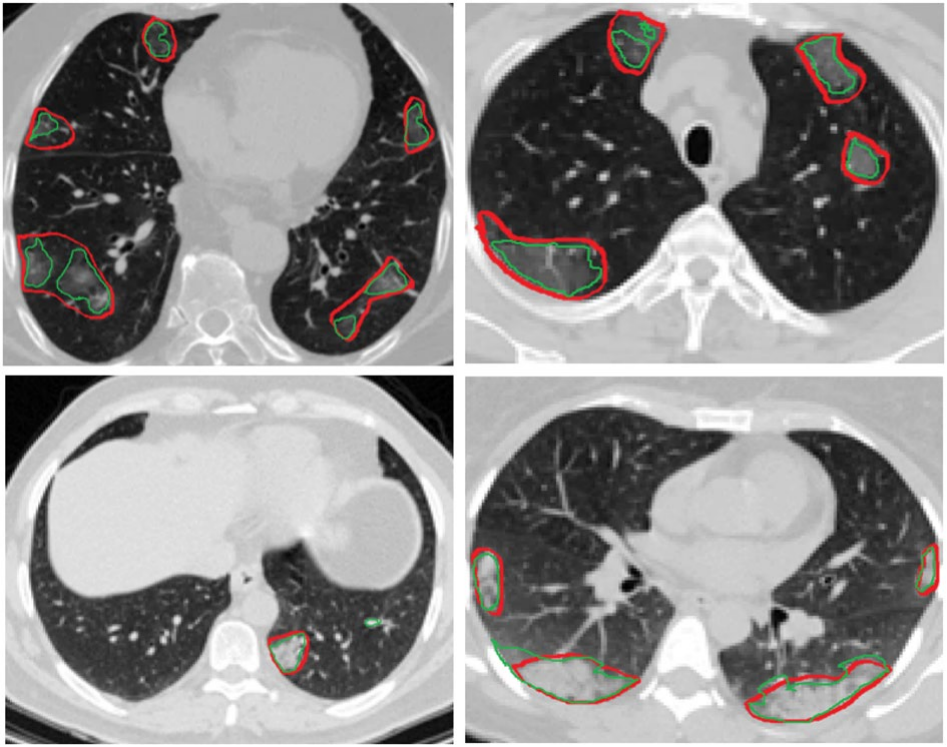
Qualitative evaluation of model performance on segmenting GGO (first row) and consolidation (second row) infection patterns. Ground truth and predicted infection regions have been indicated with red and green borders, respectively.

As mentioned previously, in the COVID-CT-Rate dataset, we mainly focused on providing lesion masks for GGO and consolidation infection patterns, as the most frequently observed COVID-19 CT manifestations. Here, we randomly selected CT samples containing GGO and consolidation from the test set to illustrate and qualitatively discuss the model’s performance and capabilities/limitations on segmenting GGO and consolidation. As it can be observed in Fig. 10, even in multifocal lung involvement conditions, the model can ideally segment GGO and consolidation infection regions. However, in the very low-intensity ranges of the GGO patterns, the predicted infection region is smaller than the ground truth label. Due to the higher intensity range, the predicted infection regions for consolidation patterns are very close to the ground truth labels. The qualitative evaluation of the results also indicates that when the infection patterns are complicated with an irregular shape, the model prediction can be more accurate than manually provided ground truth labels.

For comparative analysis, we compare the proposed $\text {COVID-Rate}$ framework with the following benchmark models: (i) Standard U-Net; (ii) Attention U-Net^37^; (iii) U-Net++^38^, (iv) Residual U-Net with CPB module, (v) $\text {COVID-Rate}$ without CPB module, and (vi) enhanced $\text {COVID-Rate}$ results via unsupervised enhancement method. It should be noted that for implementing the proposed unsupervised enhancement method, in each cross-validation, the trained model was applied to a test set containing 588 CT images from 12 COVID-19 patients acquired by the same scanner of COVID-CT-Rate dataset, for which we did not have the ground truth masks. Then, considering that we are blind to the ground truth masks, we quantified the model confidence on each predicted mask by calculating CI measure and selected the top 25%, if their CI is not less than 90% the most certain predictions. This subset of most certain predictions, including CT images and the associated predicted masks, are then used to re-train the initially pre-trained model. Table 4 presents the average of different evaluation metrics for each model. A common 10-fold cross-validation with similar data split and pre-processing steps is used to train/test different models. To examine effects of removing non-lung regions, as the main pre-processing step, on the model’s overall performance, we have trained the model without performing the lung extraction step. The model achieved 77.2%, 99.6%, 78.3% and 0.006 for the DSC, SPC, SEN, and MAE metrics, respectively. As it can be observed, the lung extraction step significantly improves the DSC and SEN metrics. The reason is that removing non-lung parts from the CT images enables the learning model to be restricted to the region of interest, reducing false-positive detections that fall outside the lung area. Another significant advantage of removing non-lung regions in the pre-processing step is that rendering all non-lung pixels to zero decreases the computational costs and helps the model converge faster.

**Table 4:** Quantitative comparison of different architectures in segmenting COVID-19 lesions. The presented results are the average of the obtained results through a 10-fold cross-validation process.

Architecture	DSC	SPC	SEN	MAE
Standard U-Net	0.7793	0.9963	0.7622	0.0059
U-Net++	0.7891	0.9964	0.8118	0.0061
Attention U-Net	0.7911	**0**.**9974**	0.7842	0.0056
Residual U-Net with CPB	0.7921	0.9968	0.8239	0.0055
$\text {COVID-Rate}$ without CPB	0.7991	0.9968	0.8296	0.0054
$\text {COVID-Rate}$	0.8036	0.9968	0.8340	**0**.**0053**
Enhanced $\text {COVID-Rate}$	**0**.**8069**	0.9969	**0**.**8354**	**0**.**0053**

The best results have been highlighted in bold.

#### Generalization: assessment on external datasets

When developing AI-based models using CT images, it is crucial to assess if the trained model can be generalized on CT images from a new scanner. This is mainly because CT images, acquired from different scanners with varying acquisition settings, show different resolutions and characteristics. For this purpose, we evaluate the $\text {COVID-Rate}$ performance on a third dataset (referred to as Dataset C) that is different from our training set and contains COVID-19 CT images from nine patients. We ignore one of the CT volumes that shows minimal infection regions (the infection rate is almost zero) and include the rest in the experiments. In the first step of this generalization assessment experiment, we examine the $\text {COVID-Rate}$ model only on CT images containing COVID-19 lesions (372 out of 787 CT images contained infection regions). The results are shown in Table 5. The DSC, SPC, SEN, and MAE metrics for the first test are 0.794, 0.993, 0.901 and 0.0087. The obtained results show that the model can reasonably work on CT images from a new scanner. We compare performance of the $\text {COVID-Rate}$ with Ref.^39^ that has used 374 infected CT slices from Dataset C along with 100 infected CT slices for training and testing their proposed segmentation network through a five-fold cross-validation. Although their model has been trained on this dataset, the proposed $\text {COVID-Rate}$ framework outperforms their model in terms of SPC and SEN metrics and yields comparable DCS results.

**Table 5:** Model evaluation on external dataset on CT images containing COVID-19 lesions and on whole CT volumes.

Method	Input data	Validation type	DSC	SPC	SEN	MAE
Ref.^40^	CTs with infection regions	Cross-validation	**0**.**831**	0.993	0.867	–
$\text {COVID-Rate}$	CTs with infection regions	External validation	0.794	0.9931	0.901	0.0087
Enhanced $\text {COVID-Rate}$	CTs with infection regions	External validation	0.806	**0**.**9933**	**0**.**914**	**0**.**0082**
Ref.^41^	Whole lung volumes	External validation	0.597	0.977	0.865	0.033
$\text {COVID-Rate}$	Whole lung volumes	External validation	0.787	0.9961	0.901	0.0049
Enhanced $\text {COVID-Rate}$	Whole lung volumes	External validation	**0**.**7998**	**0**.**9962**	**0**.**914**	**0**.**0046**

Significant values are in bold.

**Figure 11:**
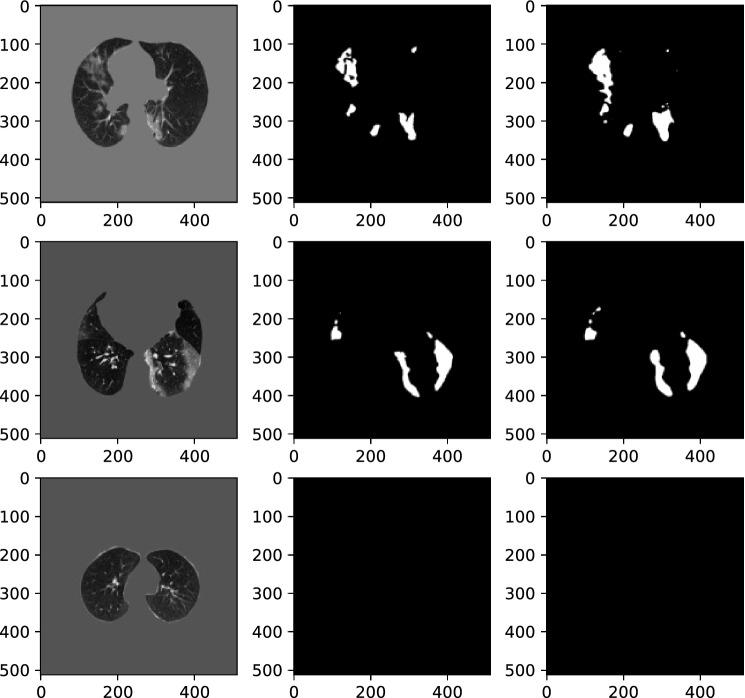
Qualitative evaluation of the model generalization on an external dataset. From left: Original image, Ground truth mask, Predicted mask. The model predicts a black mask for CT images with no trace of viral infection.

**Figure 12:**
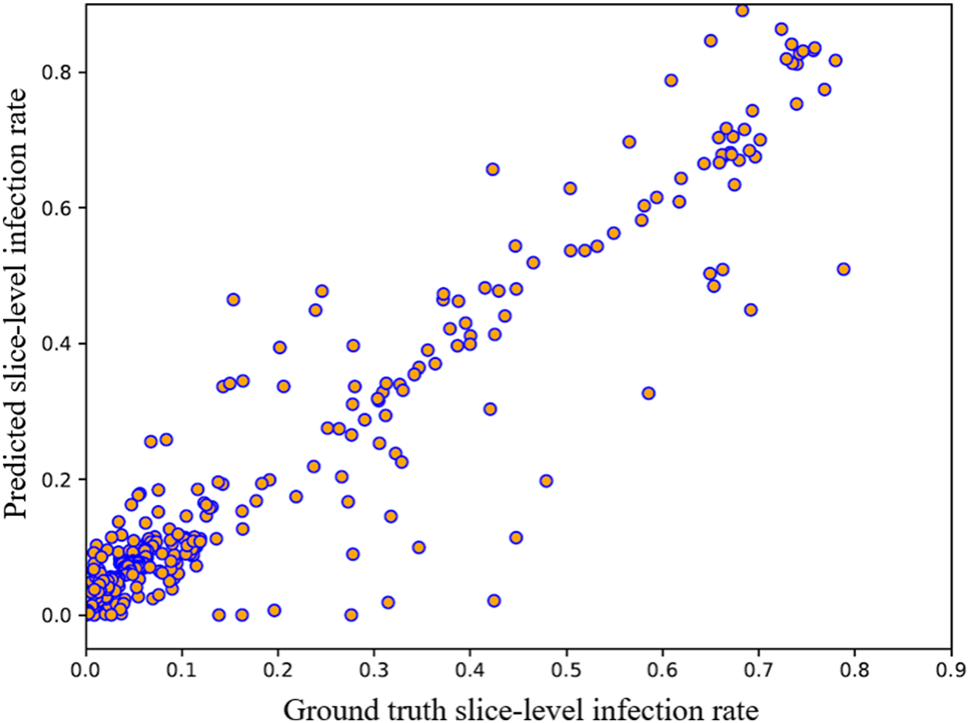
Ground truth and predicted slice-level infection rate in generalization assessment experiment.

In practical settings, COVID-19 CT volumes consist of several slices, some of them with regions of infection and the rest with no evidence of infection. To assess the model’s performance in segmenting COVID-19 lesions, we test the $\text {COVID-Rate}$ on whole lung volumes of eight COVID-19 patients (683 out of 787 CT images included lung tissues). Figure 11 demonstrates some examples of the ground truth and the predicated masks from this experiment. As can be observed, the model predicts a black mask for CT images with no trace of infection. The experiment results over the 683 test images containing lung tissues are 0.787, 0.9961, 0.901 and 0.0049 for DSC, SPC, SEN, and MAE metrics. Compared to Ref.^40^, which have used 638 slices of this dataset (285 normal slices and 353 slices with COVID-19 lesions) as an external test set for evaluation of their semi-supervised segmentation framework, the $\text {COVID-Rate}$ achieved better results across all the evaluation metrics. The mean absolute error between the ground truth and predicted infection rate for each CT image is 0.049 and 0.0087 for CT images with and without evidence of infection, respectively. Figure 12 represents the linear relationship between ground truth and predicted infection rates for CT images containing COVID-19 lesions. The Pearson correlation coefficient between the two groups of data is measured 0.959 indicating that the predicted and ground truth infection rates are highly correlated. In the next step, we examined the effectiveness of our proposed unsupervised enhancement method on improving model generalization. For this purpose, we applied our trained model to the infected slices of the external dataset. Then, we measured the model confidence on the predicted mask using the introduced CI and selected the top 25%, if their measured CI is not less than 0.9, as the most confident prediction. The most confident prediction and their predicted infection masks were used to retrain our initially trained models. The performance of the enhanced model was then examined on (i) the infected slices and (ii) whole CT volumes (CT images containing lung regions), and the results were presented in Table 5. As can be observed, the unsupervised enhancement method could improve the results regarding all evaluation metrics. This is because the initial model has no insight into the characteristics of the CT images from the external dataset. The enhancement approach gives the initially trained model some helpful information on the new dataset, reducing the shifts between the original training set and the external dataset, leading to improved performance. Indeed, the obtained results demonstrate the enhancement method’s capability in improving the model’s generalization upon receiving a new dataset from a different scanner, addressing a critical challenge in applying AI models in medical imaging.

#### Model evaluation on discriminating CT images with infection

To further explore how the proposed $\text {COVID-Rate}$ framework performs on whole CT volumes, we use a subset of Dataset D that contains 50 CT volumes with slice-level labels and evaluate the $\text {COVID-Rate}$ performance on discriminating infected CT images from non-infected ones. According to our framework, we first extracted lung regions from CT images. From 6846 CT images containing lung tissues, 3503 showed COVID-19 lesions. Since our $\text {COVID-CT-Rate}$ dataset is a subset of Dataset D, we eliminated the 267 common CT slices that were overlapping between the two datasets. The rest of the CT images, a total of 6579 images from 50 patients, were passed through the segmentation network. The model predicts a mask containing lesion regions for CT images with infection and a black mask for CT images without infection. To avoid spurious effects from minor imaging findings, we consider a CT image as an “infected image” only if its predicted infection rate is more significant than 0.005. The accuracy, sensitivity, and positive predictive value for this discrimination experiment are 0.877, 0.946 and 0.831, which are promising for the potential application of the $\text {COVID-Rate}$ framework on whole lung volumes.

#### Efficacy of the proposed synthetic data augmentation

**Figure 13:**
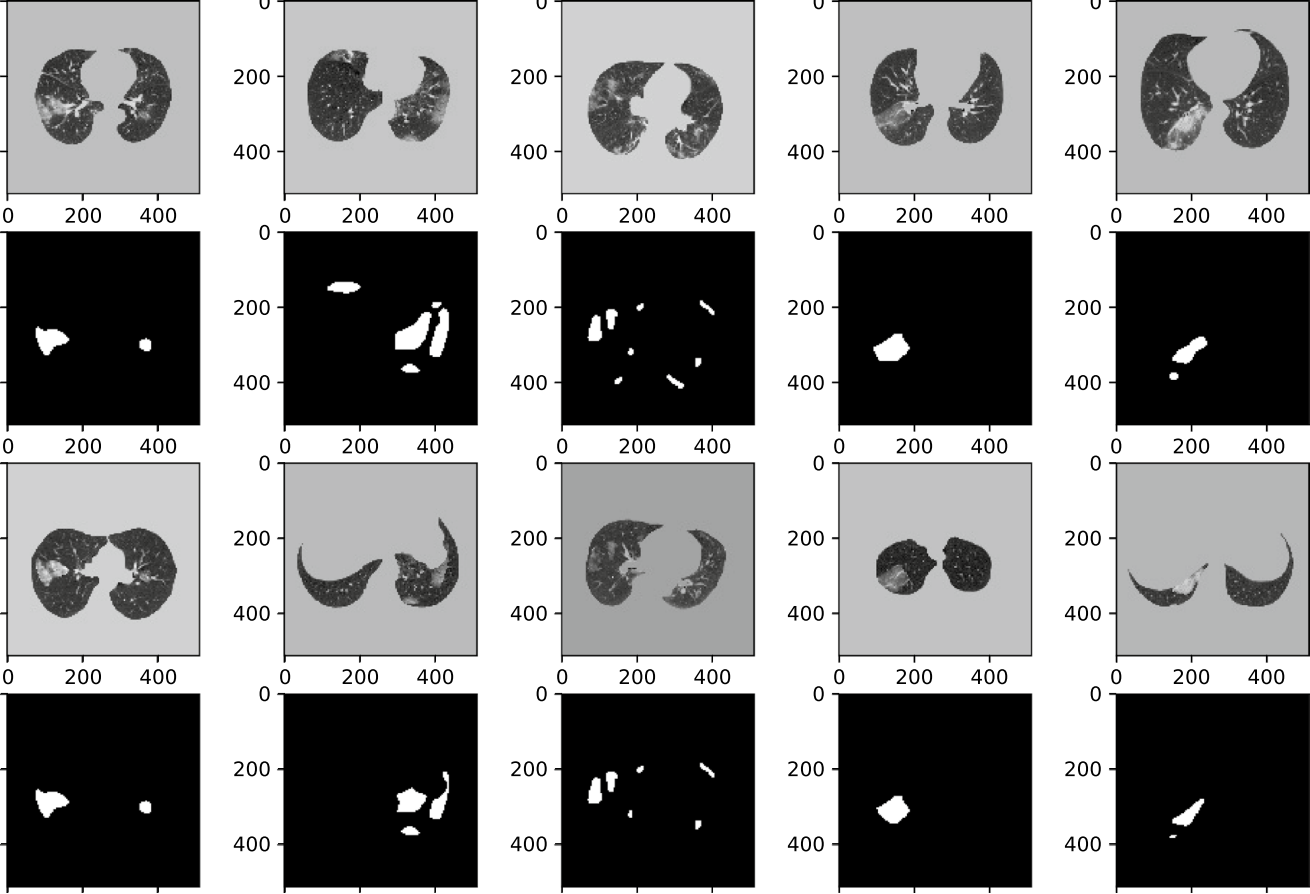
Samples of synthetic images and infection masks. First and second rows: COVID-19 infected CT images and their corresponding infection masks. Third and fourth rows: Synthetic images and the adjusted infection masks.

To explore the efficacy of our proposed data augmentation technique in the training process, first, we trained the $\text {COVID-Rate}$ model without the CPB module on the COVID-CT-Rate dataset. We split the dataset into three subsets for training (60%), validation (10%), and testing (30%). The conventional data augmentation techniques, including zooming, shifting, and shearing, were used during the training. In the next step, we performed the same experiment using a training set augmented by our data augmentation technique. For this purpose, we used 988 CT images from nine healthy lung volumes acquired by the same scanner of the COVID-CT-Rate. In each fold of the cross-validation, we generated synthetic pairs of CT images and infection masks based on the training set of each fold. We then eliminated the synthetic images with an infection rate of less than 0.01. To investigate the effect of incorporating different numbers of synthetic images with the training set, we performed a set of experiments with the number of synthetic images equal to 0.5*N*, 1*N*, 1.5*N*, and 2*N*, where *N* is the size of the original training set. In each fold, the validation and test sets are kept the same as the previous step of the experiment. The results of both experiments through 10-fold cross-validation are presented in Table 6. As demonstrated, the model yields the optimum results when the number of synthetic images is about to *N*. Indeed, this method of generating synthetic images introduces more variability than the conventional data augmentation methods and can considerably improve the results. However, by increasing the number of synthetic images, the infection patterns with the same intensity range will be repeated during the training, causing the overfitting problem. Figure 13 demonstrates some samples of the synthetic images and their corresponding infection masks. The synthetic images with the infection rate more significant than 0.01 are concatenated with the training set.

**Table 6:** Evaluation of the proposed data augmentation method’s efficacy through a two-step 10-fold cross-validation approach.

Training set	Number of synthetic images	DSC	SPC	SEN	MAE
		Ave ± std	Ave ± std	Ave ± std	Ave ± std
Existing training set	–	$0.7624 \pm 0.02$	$0.9944 \pm 0.002$	$0.8342 \pm 0.06$	$0.0079 \pm 0.001$
Augmented training set	$0.5 \times N$	$0.7696 \pm 0.02$	$0.9944 \pm 0.001$	$0.8573 \pm 0.04$	$0.0077 \pm 0.001$
	$1 \times N$	$0.782 \pm 0.01$	$0.9948 \pm 0.001$	$0.8614 \pm 0.02$	$0.0073 \pm 0.001$
	$1.5 \times N$	$0.7604 \pm 0.02$	$0.9944 \pm 0.001$	$0.8393 \pm 0.05$	$0.0079 \pm 0.001$
	$2 \times N$	$0.752 \pm 0.05$	$0.9953 \pm 0.001$	$0.79 \pm 0.1$	$0.0077 \pm 0.001$

#### Capsule-network-based classifier integrated with the COVID-Rate segmentation network

**Figure 14:**
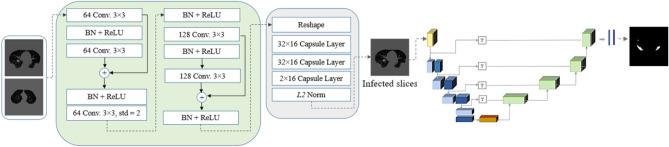
The structure of the Capsule-network-based classifier integrated with the COVID-Rate segmentation network.

As another variation of our COVID-19 lesion segmentation framework, as illustrated in Fig. 14, we embedded a capsule-network-based module prior to the segmentation network to identify CT images containing COVID-19 manifestations from the non-infected ones. The CT images identified as infected slices will then be fed into the segmentation network for the lesion segmentation task. The classifier module takes the lung area of 2D CT images as the input and predicts a label demonstrating whether the given image is infected or not. Capsule networks^41^, which have demonstrated superior capability in learning informative features from small datasets, form the basis of the developed classifier. An iterative process, known as “Routing by Agreement”, is used in capsule networks, helping identify the spatial relation between extracted features from an image. The detailed structure of the capsule-network-based module is a modified variation of the model architecture proposed in^34^. Specifically, the classifier adopts five convolution layers, followed by a BN layer and the ReLU activation function. Instead of the max-pooling layer, we utilize a $3 \times 3$ convolution layer with stride 2 to mitigate information loss during downsampling. In addition, residual connections facilitate the model’s convergence during the training process. The fifth convolution layer’s output is then reshaped and fed into the three consecutive capsule layers. The last capsule layer includes two capsules determining the probability of class labels. The candidate images from the classifier are then used as input to the segmentation network to generate the infection masks. In this experiment, we first measured the performance of our previously trained COVID-Rate segmentation network on only COVID-Rate-CT images, where the model achieved a DSC of 0.781, SPC of 0.9961, SEN of 0.812, and MAE of 0.0066. Then, for training the two-stage framework, we used 433 infected CT images from the COVID-CT-Rate dataset integrated with 988 normal CT images from nine patients acquired by the same scanner. We randomly split the dataset into 60%, 10% and 30% for training, validation, and test sets. The training set, containing infected and non-infected slices, was used as the classifier’s input. The candidate CT images, predicted as the infected class, were fed into the segmentation network to produce the infection masks. In each cross-validation, we used the weights of the previously trained segmentation network as the initial weights and let the model’s weights be updated during the training process. The integrated framework achieved a DSC of 0.767, SPC of 0.9963, SEN of 0.781, and MAE of 0.0068. Although the integrated framework yields lower performance than the segmentation network being trained only on the infected slices, the obtained results are promising for applying the COVID-Rate segmentation network with integrated Capsule-network-based classifier in scenarios without availability of experts for image-level labeling of CT images.

## Discussion

This study is motivated by the urgent quest to develop accurate and reliable automated models for prognostic assessment of COVID-19 pneumonia coupled with the need for providing high-quality COVID-19 lesion segmentation datasets. To better position this study within the existing literature, first, we discuss the related works.

**Related works:** Recently, several autonomous models are proposed/designed based on DL solutions to assist in the rapid diagnosis of COVID-19 from other types of respiratory infections^,42−50^. There are, however, fewer works on developing DL-based models for segmentation and quantification of COVID-19 lesions. Segmentation models designed based on DL models are mainly developed based on CNNs. Such DL models are image-to-image networks containing an encoding path for extracting high-resolution features from input images and a decoding path for generating masks indicating the regions of interest. The majority of the COVID-19 lesion segmentation models have been developed upon U-Net^23^ due to its superiority for the task of medical image segmentation^51,52^. For example, using a U-Net architecture integrated with DenseNet blocks in the encoder path, Chaganti et al.^14^ proposed a segmentation model to quantify lung abnormalities caused by COVID-19 from CT images. Similarly, Zhou et al.^39^ proposed an enhanced U-Net segmentation model by incorporating spatial and channel attention mechanisms. Segmentation networks can be designed based on 2D or 3D CNNs to segment COVID-19 regions of infection either on slice-level or patient-level basis. It should be noted that 3D segmentation networks that can segment infections on the whole lung volumes are more desired from practical point of view. However, they need a large amount of 3D annotated lung volumes for efficient training and are more computationally expensive. Ref.^52^ trained both 2D and 3D variants of the U-Net model for segmenting COVID-19 infections from chest CT images of 558 patients confirmed with COVID-19 pneumonia. According to their experiments, the 2D U-Net model achieved a Dice Similarity Coefficient (DSC) of 79.07% while 3D U-Net obtained DSC of 70.35%, demonstrating that on small datasets, slice-level segmentation can achieve better results.

Generally speaking, to efficiently train a DL-based segmentation model, large amount of annotated CT images are required. However, due to nonuniform contrasts and irregular boundaries of COVID-19 lesions, providing pixel-level labeled datasets is challenging and expensive. Multi-task DL networks that jointly perform COVID-19 classification and lesion segmentation have shown promising results when facing lack of annotated data^53,54^. In this context, Wu et al.^54^ developed a joint classification and segmentation network that distinguishes COVID-19 cases from uninfected ones and at the same time produces the infection masks. They incorporated two parallel branches to extract image features for classification and segmentation tasks separately. The extracted features are then combined and fed into a decoding path to generate COVID-19 lesion masks. Performing classification and segmentation tasks simultaneously provides deep supervision for training the network on image-level and pixel-level, yielding more accurate results on both tasks. Transfer learning is a powerful technique to resolve shortage of data when deep learning models are designed for medical image analysis tasks with limited labeled datasets. Existing DL models for COVID-19 lesion segmentation have taken advantage of both natural images (like the ImageNet dataset) and medical images to enhance their model’s performance. The use of top-performing CNN models such as ResNet, DenseNet, and Inception-Net pre-trained on ImageNet dataset as the backbone of encoding path can improve the segmentation results^55,56^. For instance, Ref.^57^ leverages a two-stage transfer learning approach to deal with the lack of annotated data. In the first step, as the basis for their segmentation network’s encoding path, they utilize ResNet50 trained on ImageNet. The next step involves performing another transfer learning stage and pre-training the segmentation network using a large chest CT dataset for lung nodule detection. The second stage compensates for the large gap between the natural and COVID-19 chest images and enhances the final training on COVID-19 chest CT images. Some research studies use segmentation methods that require less data for training to overcome the lack of sufficient pixel-level annotated CT images. Authors in Ref.^40^ developed a semi-supervised segmentation framework that is trained only on 50 annotated CT images of COVID-19 patients together with 1600 unlabeled CT images. Their proposed model segments the COVID-19 lung abnormalities under both one and two classes of infections. Integrating semi-supervised and few-shot learning methods, Ref.^58^ introduced a segmentation network that can learn from a few number of labeled chest CT images. Authors proposed a dual-path network architecture for few-shot learning and incorporated an adaptive knowledge exchange module between the networks’ paths to enhance the model’s performance in segmenting COVID-19 lesions. Considering COVID-19 classification as a few-shot learning problem and leveraging a contrastive learning approach, Ref.^59^ trained an encoder that can capture discriminative feature representations from open-access datasets. The prototypical network is then adopted to detect COVID-19 cases from CT images. Alternatively, one may choose to train a DL network with weak supervision through lower-quality labels, which are inexpensive and need less time to be generated. For instance, Laradji et al.^60^ proposed a weakly supervised learning method by annotating a single pixel for any COVID-19 infection region on a CT image and achieved a DSC range of 68–75% on three public datasets. Their proposed labeling scheme reduced the annotation time for each infection region from 10–15 s to 1–3 s. Using some simple operations, Yao et al.^61^ synthesized COVID-19 regions of infection on chest CT images of healthy people and generated fake image-label pairs for training their segmentation network with no labeled data. It is worth mentioning that although these studies suggest effective solutions in compensating the lack of sufficient labeled data, they cannot yield the same success of fully supervised learning methods with accurate labels. This study aimed to address this gap.

**The** $\text {COVID-Rate}$ **Framework:** Capitalizing on the above discussion, we proposed a deep convolutional neural network model, the socalled $\text {COVID-Rate}$ framework, for segmenting COVID-19 legions from chest CT images. A high-quality COVID-19 segmentation dataset containing 433 CT slices from 82 patients is also introduced.

Several comprehensive experiments were conducted to evaluate efficacy and limitations of the proposed $\text {COVID-Rate}$ model on 2D CT images and whole CT volumes, using both internal and external datasets. The results indicate that $\text {COVID-Rate}$ can efficiently segment COVID-19 regions of infection from CT images on both slice-level and patient-level basis. To cope with data hungry nature of deep AI models, a novel data augmentation method is proposed that generates synthetic CT images and infection masks by inserting regions of infection from COVID-19 infected CT images to healthy CT images. In particular, based on the results of the comparison study (Table 4), it can be observed that the standard U-Net network achieved the lowest performance in our experiments. This is mainly because that the other networks are improved variations of the U-Net. The $\text {COVID-Rate}$ network outperforms the U-Net++ and Residual U-Net with CPB across all evaluation metrics and against Attention U-Net across the DSC, SEN, and MAE metrics. The results demonstrate that incorporating CPB module can improve the model performance. Finally, the unsupervised enhancement method could improve results of the DSC, SPC, and SEN metrics while achieving the same value on the MAE metric. The results of the generalization experiment (Figs. 11 and 12) illustrate that despite being trained only on CT images with infection, our proposed $\text {COVID-Rate}$ can optimally segment COVID-19 lesions on whole CT volumes. Indeed, by quantifying the regions of infection on 2D CT slices and summing them up for the entire lung volume, assuming that the changes over a thick CT slice are negligible, the model can approximate the patient-level infection rate in COVID-19 patients.

Experimental results show that the proposed unsupervised enhancement method can improve model’s performance. However, the external test set results showed more improvement than utilizing an extra subset acquired by the same scanner used for the initial training. The main reason is that due to prior exposure to CT images from that scanner, the model did not retain much information during the retraining process on most-certain predictions. Conversely, when adopting the proposed enhancement method on an external dataset, the model does not have any information about the unseen data from a different scanner. In this way, the model was able to acquire practical knowledge about the specifications of the unseen images through the retraining process, resulting in further improvements. As a result, the proposed unsupervised method could bridge the gap between the training and independent sets, leading to an increased generalization capability. The presented results in Tables 4 and 5 further support this notion. Finally, from the results of the experiment conducted to evaluate efficacy of the proposed synthetic data augmentation mechanism (Fig. 13 and Table 6), it can be observed that model yield the optimum results when the number of synthetic images incorporated with the original dataset is equal to *N* (*N* is the size of original training set), improving the average DSC from 0.7624 to 0.782, the average SEN from 0.8342 to 0.8614, the average SPEC from 0.9944 to 0.9948, and the average MAE from 0.0079 to 0.0073. The experimental results indicate that concatenating the synthetic images generated by our data augmentation method will enrich the training set by introducing more variability to the training set, resulting in enhanced model performance. However, as the number of synthetic images increases, infection patterns following the same intensity range will be repeated during the training process, leading to overfitting.

Segmenting COVID-19 lung abnormalities under different classes of infection can yield helpful information on disease severity and stage. However, our experiments and provided CT data are limited to segmenting lung lesions under one class of data. One direction for future research is extending the dataset to model multi-class segmentation of COVID-19 lesions. Although the generalization test demonstrates that the model can work reasonably on a patient-level basis, the patients’ number in the test set is limited. More experiments on larger datasets are required to further evaluate reliability of the model patient-level application. Finally, chest CT scans can be utilized for diagnosing and assessing COVID-19 disease, however, it has been shown that standard-dose CT scanning can be highly radiation-intensive for patients, especially when multiple scans are required. In this regard, replacing low- and extra-low-dose CT scans is a safer option for the patients. Developing segmentation networks and datasets on low- and extra-low-dose CT images is another direction for future research. Segmentation models are used as the first step of severity assessment and prognosis prediction of COVID-19 patients, which would help optimize resource allocation and patient management. Future directions include extending the $\text {COVID-Rate}$ for quantifying specific COVID-19 severity measures and integrating it into a hybrid deep learning model to detect high-risk COVID-19 patients and predict adverse outcomes based on CT images and clinical/laboratory information.

## Data Availability

The Datasets and codes generated and/or analyzed during the current study are available for public access through Figshare^62^ and^63^, respectively.
